# Coexisting crystal and liquid-like properties in a 2D long-range self-consistent model

**DOI:** 10.1038/s41598-018-33889-8

**Published:** 2018-10-25

**Authors:** J. M. Maciel, M. A. Amato, M. -C. Firpo

**Affiliations:** 1 Campus Paranavaí, Instituto Federal do Paraná, 87703-536 Paranavaí, PR Brazil; 2 Instituto de Física, Universidade de Brasília, CP 04455, 70919-970 Brasília, Brazil; 3 International Center for Condensed Matter Physics, Universidade de Brasília, CP 04455, 70919-970 Brasília, Brazil; 4 LPP, CNRS, Ecole Polytechnique, Université Paris-Saclay, PSL Research University, 91128 Palaiseau, France

## Abstract

A two-dimensional class of mean-field models serving as a minimal frame to study long-range interaction in two space dimensions is considered. In the case of an anisotropic mixed attractive-repulsive interaction, an initially spatially homogeneous cold fluid is dynamically unstable and evolves towards a quasi-stationary state in which the less energetic particles get trapped into clusters forming a Bravais-like lattice, mimicking a crystalline state. Superimposed to this, one observes in symplectic numerical simulations a flux of slightly more energetic particles channeling through this crystalline background. The resultant system combines the rigidity features of a solid, as particles from a displaced core are shown to snap back into place after a transient, and the dynamical diffusive features of a liquid for the fraction of channeling and free particles. The combination of solid and liquid properties is numerically observed here within the classical context. The quantum transposition of the model may be experimentally reached using the latest ultracold atoms techniques to generate long-range interactions.

## Introduction

The present study deals with the phenomenology of a self-consistent long-range, mean-field, *N*-body system of particles in the low temperature limit, yet in the classical frame. The fact that the model is utterly devoid of quantum aspects may not prevent it from being of some relevance to understand some properties of a physical system at low temperatures. Indeed, one could argue on a general basis that the transition line between the quantum and classical worlds is rather diffuse. For instance, it has been shown very recently^1^ that strong coupling in light-matter interaction with large amounts of particles, a phenomena that was previously thought to be of quantum nature, could be explained classically. One may also recall that there have been attempts to describe the phenomenon of supersolidity in the classical mechanical hydrodynamics^2^. Long-range refers here to interactions decaying as *r*^−*α*^ at large distance *r* with 0 ≤ *α* ≤ *D*, with *D* the space dimension.

The model studied here may be described by a set of a large number *N* of particles interacting through mean-field forces, having initially zero temperature and zero total momentum and being initially homogeneously distributed in the two-dimensional space. It is shown that this model evolve self-consistently to an out-of-equilibrium quasi-stationary state (QSS) and gives rise to a space-modulated particle density. This feature defines a crystal, through which some subset of the most energetic particles flows so that the momentum of this particle subset compensates the crystal momentum. The fact that this state is out-of-equilibrium relates to the well-known ergodicity breaking features of mean-field collisionless (Vlasov) systems. The study of the scaling with *N* of the lifetimes of the QSS in long-range systems has been an active research field (See e.g.^3–9^). The fact that these lifetimes diverge with *N* means that those systems may practically never reach the Gibbs-Boltzmann thermodynamic equilibrium justifying their treatment in the nonequilibrium context e.g. with effective macro-particle and collective modes low-dimensional models^10^, core-halo descriptions^11^ or alternative, non-Gibbsian, thermostatistics^12,13^. For quantum systems, a similar divergence of equilibration times with the system size, *N*, was reported for long-range quantum spin models^14^.

The model is the 2*D*-Hamiltonian Mean Field (HMF) model with mixed, attractive and repulsive, infinite-range interactions. Interestingly, the (*α* = 0) HMF model was shown to display features universal to systems with 0 ≤ *α* ≤ *D*/2 interaction range^15–17^ enforcing its status of paradigm for long-range systems. In a previous work on the 2*D*-HMF^18^, we numerically demonstrated on microcanonical Monte Carlo simulations that, at minimal energy densities, the system organizes in a Bravais-like lattice forming cores playing the role of the atoms in a crystal structure. In the present study, the energy density of the system is not minimal yet the initial temperature is vanishingly small and we use molecular dynamics simulations. As the system follows its natural evolution, it slightly warms up and organizes into a ‘cold’ crystal structure that is gone through by a coherent flux of the most energetic particles. After introducing the physical model, evidence is given of the dual solid and liquid nature of the quasi-stationnary state. Its quantum realization could be produced with cold atoms driven by laser light where multiple scattering of photons by atoms gives rise to infinite-range forces when the atoms couple to a single mode high-finesse cavity^19^.

## The Two-Dimensional Hamiltonian Mean-Field Model

### Derivation

The 2*D*-Hamiltonian Mean Field (HMF) model was first proposed by Antoni and Torcini^20,21^ as a two dimensional generalization of the Hamiltonian Mean Field (HMF) model in the fully attractive case for the study of *N*-body self-gravitating systems. A generic two-body potential in a two dimensional square box of side 2*π* with periodic boundary conditions can be Fourier expanded as

1 $$V(x,\,y)=\sum _{{\bf{k}}=({k}_{x},\,{k}_{y})}\,\hat{V}({\bf{k}}){e}^{i{\bf{k}}\cdot {\bf{r}}}.$$

Retaining only the most long-range terms, with |**k**| = 1 and $|{\bf{k}}|=\sqrt{2}$, in the expansion and requiring that the potential be invariant under rotations of multiples of *π*/4^22^ yields the following truncation of the potential in Eq. (1)

2 $$V(x,\,y)=a-c(\cos \,x+\,\cos \,y)-d\,\cos \,x\,\cos \,y,$$

where *a* is an arbitrary scaling constant, *c* and *d* are coupling constants, and due to the rotation invariance *c* is related to the energy scaling and *d* is the only free parameter^22^.

Considering *N* particles interacting through the potential in Eq. (2) and setting 2*c* + *d* = −*a*, one gets the following Hamiltonian

3 $$H=K+V$$

with

4 $$K=\sum _{i=1}^{N}\,{K}_{i}=\sum _{i=1}^{N}\,\frac{{p}_{ix}^{2}+{p}_{iy}^{2}}{2},$$

and

5 $$V=\frac{1}{2N}\sum _{i,j=1}^{N}\{c\mathrm{[2}-\,\cos \,({x}_{i}-{x}_{j})+\,\cos \,({y}_{i}-{y}_{j})]+d\mathrm{[1}-\,\cos \,({x}_{i}-{x}_{j})\,\cos \,({y}_{i}-{y}_{j}\mathrm{)]\}.}$$

Here the *p*_*ix*_ and *p*_*iy*_ are the conjugate momenta to the space positions *x*_*i*_ and *y*_*i*_. The 1/*N* prefactor corresponds to the Kac’s prescription^23^. It recovers the extensivity of the pair potential and is equivalent to a time rescaling of the type $t^{\prime} =\sqrt{N}t$. The first term in Eq. (5) is the potential of two uncoupled (one-dimensional) HMF models, while the second term couples the *x* and *y* directions.

Defining the four self-consistent mean-field variables as

6 $${{\bf{M}}}_{{\bf{1}}}=({\langle \cos x\rangle }_{N},{\langle \sin x\rangle }_{N})={M}_{1}(\cos \,{\psi }_{1},\sin \,{\psi }_{1}),$$

7 $${{\bf{M}}}_{{\bf{2}}}=({\langle \cos y\rangle }_{N},\,{\langle \sin y\rangle }_{N})={M}_{2}(\cos \,{\psi }_{2},\,\sin \,{\psi }_{2}),$$

8 $${{\bf{P}}}_{+}=({\langle \cos (x+y)\rangle }_{N},\,{\langle \sin (x+y)\rangle }_{N})={P}_{+}(\cos \,{\psi }_{+},\,\sin \,{\psi }_{+}),$$

9 $${{\bf{P}}}_{-}=({\langle \cos (x-y)\rangle }_{N},\,{\langle \sin (x-y)\rangle }_{N})={P}_{-}(\cos \,{\psi }_{-},\,\sin \,{\psi }_{-}),$$

with **M**_**1**_ and **M**_**2**_ playing the role of the magnetization fields and **P**_+_ and **P**_−_ of the polarization fields and making use of the notation 〈⋅〉_*N*_ for the average over the *N* particles, the Hamiltonian in Eqs (3–5) simply reads

10 $$H=K+N\frac{c}{2}\mathrm{(2}-{{M}_{1}}^{2}-{{M}_{2}}^{2})+N\frac{d}{4}\mathrm{(2}-{{P}_{+}}^{2}-{{P}_{-}}^{2}\mathrm{).}$$

The dynamics of any single particle *i* can be easily shown to obey the equations of motion

11 $${\ddot{x}}_{i}=c{F}_{\mathrm{1,}i}+\frac{d}{2}({F}_{+,i}+{F}_{-,i}),$$

12 $${\ddot{y}}_{i}=c{F}_{\mathrm{2,}i}+\frac{d}{2}({F}_{+,i}-{F}_{-,i}),$$

using the notations

13 $${F}_{\mathrm{1,}i}=-\,{M}_{1}\,\sin ({x}_{i}-{\psi }_{1}),$$

14 $${F}_{\mathrm{2,}i}=-\,{M}_{2}\,\sin ({y}_{i}-{\psi }_{1}),$$

15 $${F}_{+,i}=-\,{P}_{+}\,\sin ({x}_{i}+{y}_{i}-{\psi }_{+}),$$

16 $${F}_{-,i}=-\,{P}_{-}\,\sin ({x}_{i}-{y}_{i}-{\psi }_{-}\mathrm{).}$$

At any time *t*, the equations (11 and 12) are obtained from the following one-particle Hamiltonian (defined up to a constant of integration *C*)

17 $$\begin{array}{lll}h({{\bf{p}}}_{i},\,{{\bf{r}}}_{i},\,t) & = & \frac{{p}_{ix}^{2}+{p}_{iy}^{2}}{2}+c\mathrm{\{2}-{M}_{1}(t)\,\cos \,[{x}_{i}-{\psi }_{1}(t)]-{M}_{2}(t)\,\cos \,[{y}_{i}-{\psi }_{2}(t)]\}\\  &  & +\frac{d}{2}\mathrm{\{2}-{P}_{+}(t)\,\cos \,[{x}_{i}+{y}_{i}-{\psi }_{+}(t)]-{P}_{-}(t)\,\cos \,[{x}_{i}-{y}_{i}-{\psi }_{-}(t)]\}+C\mathrm{.}\end{array}$$

The constant of integration can be fixed by using the conservation of the total energy yielding

18 $$\sum _{i=1}^{N}\,h({{\bf{p}}}_{i},\,{{\bf{r}}}_{i},\,t)=\sum _{i\mathrm{=1}}^{N}\,h({{\bf{p}}}_{i},\,{{\bf{r}}}_{i},\,\mathrm{0)}\equiv {U}_{0}\mathrm{.}$$

There is another constant of motion in this system: the total momentum

19 $${\boldsymbol{\Pi }}=\sum _{i=1}^{N}\,{{\bf{p}}}_{i}.$$

In the numerical simulations, this is taken to be identically zero.

### Equilibrium and out-of-equilibrium emergence of a Bravais lattice structure

The phase space trajectories of a Hamiltonian system such as Eq. (10) are constrained on a constant energy surface in phase space. Consequently, the time averages computed from the numerical solutions of the equations of motion are expected to converge to microcanonical ensemble averages. For some long-range interacting systems, the ensembles may not be equivalent and so it turns out that the microcanonical ensemble is the natural ensemble to derive equilibrium statistical mechanics.

**Figure 1:**
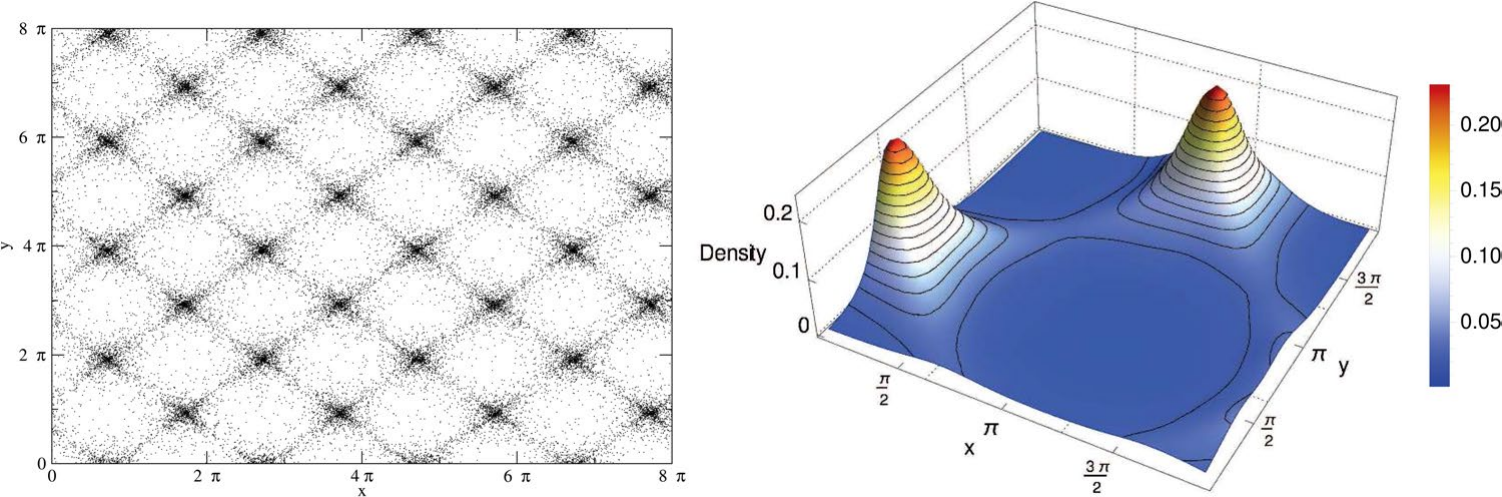
(left) Periodic Bravais-like structure in the mixed attractive/repulsive 2*D*-HMF model. A flux of particles is hopping from one core to another for the energy density *H*/*N* close to −0.5. The parameters were fixed to *c* = −1 and *d* = 1 in Eq. (5). (right) Space distribution of the particle density in an elementary [0:2*π*] × [0:2*π*] pattern.

In a previous work^18^, we studied the mixed case with attractive polarization mean-fields and repulsive magnetization mean-fields by choosing *c* = −1 and *d* = 1. Due to the invariance of the Hamiltonian under rotations by multiples of *π*/4, a simple interchange in the “charge” of the fields would provide the same results. The equilibrium statistical mechanics studies led to the following results obtained in the microcanonical ensemble: along the repulsive direction, the system behaves as the antiferromagnetic-like HMF model, whereas along the attractive directions, it behaves as the ferromagnetic-like HMF model, except that for the low energy phase, it exhibits a bicluster, instead of a single cluster structure. Due to the periodicity of the potential, one can increase the space period length by a multiple of 2*π*, and turn the bicluster into a periodic structure which can be regarded as a Bravais-like lattice. This space ordering into a Bravais lattice structure is not only present in the equilibrium states but also in the quasi-stationary states (QSS). Indeed, since the model (10) is long-range, its dynamics, computed here using a fourth-order symplectic scheme^24^, exhibits ergodicity breaking features. As already observed, e.g. in the HMF model, after an initial phase of violent relaxation, the system settles in an out-of-equilibrium quasi-steady state, the lifetime of which diverges with *N*. Figure 1 displays the typical Bravais lattice structure emerging in the QSS phase starting from cold and space homogeneous initial conditions. Contrarily to Monte Carlo simulations^18^ that do not capture the real dynamics of the system, in molecular dynamical simulations one observes that some particles are hopping from one cluster to the others so that there is a flux of particles hopping from one cluster to another, the amount of the flux depending on the system energy.

We shall now report the results on the formation of the Bravais lattice structure and QSS’s evolution and characteristics in the mixed attractive/repulsive 2*D*-HMF model.

## Dynamical Features of the Attractive/Repulsive 2*D*-HMF Model in the Low-Temperature Regime

### Initial conditions

Our previous results on this system^18^ showed that the homogeneous distribution of a cold ensemble of particles is linearly unstable in the attractive directions and linearly stable in the repulsive directions. The resulting linear instability triggers the so-called violent relaxation process, according to Lynden-Bell’s wording^25–27^. The initial distribution functions considered in the present analysis are the following water-bag distribution functions

20 $$\begin{array}{rcl}{f}_{0}({\bf{r}},{\bf{p}}) & = & \frac{1}{{\mathrm{(4}\pi n{\rm{\Delta }}p)}^{2}}[{\rm{\Theta }}(x)-{\rm{\Theta }}(x-2n\pi )]\\  &  & \times [{\rm{\Theta }}(y)-{\rm{\Theta }}(y-2n\pi )]\,{\rm{\Theta }}({\rm{\Delta }}p-|{p}_{x}|)\,{\rm{\Theta }}({\rm{\Delta }}p-|{p}_{y}|),\end{array}$$

where *r* ≡ (*x*, *y*), **p** ≡ (*p*_*x*_, *p*_*y*_) and Θ is the Heaviside unitary step function. Inasmuch as the mean square momentum for distribution (20) is given by

21 $${\langle {p}^{2}\rangle }_{t=0}=\int \,d{\bf{p}}d{\bf{r}}{p}^{2}{f}_{0}({\bf{r}},{\bf{p}})=\frac{\mathrm{2(}{\rm{\Delta }}p{)}^{2}}{3},$$

the distribution is considered to be an ensemble of cold particles if Δ*p* is chosen to be sufficiently small. The total energy is given by

22 $$U=N\frac{\langle {p}^{2}\rangle }{2}+\frac{N}{2}[-1+({M}_{1}^{2}+{M}_{2}^{2})-\frac{1}{2}({P}_{+}^{2}+{P}_{-}^{2})]\mathrm{.}$$

In the present Hamiltonian system, the energy is conserved and equal to

23 $${U}_{0}=N[\frac{{({\rm{\Delta }}p)}^{2}}{3}-\frac{1}{2}],$$

that is its value at time *t* = 0. For future reference we set Δ*p* = 10^−6^ in this work. The fourth-order symplectic integrator^24^ ensures that the relative error in energy remains of the order ${\rm{\Delta }}U/U={\mathscr{O}}{\mathrm{(10}}^{-12})$ in the numerical results presented here.

### Characteristics of the quasi-stationary states

**Figure 2:**
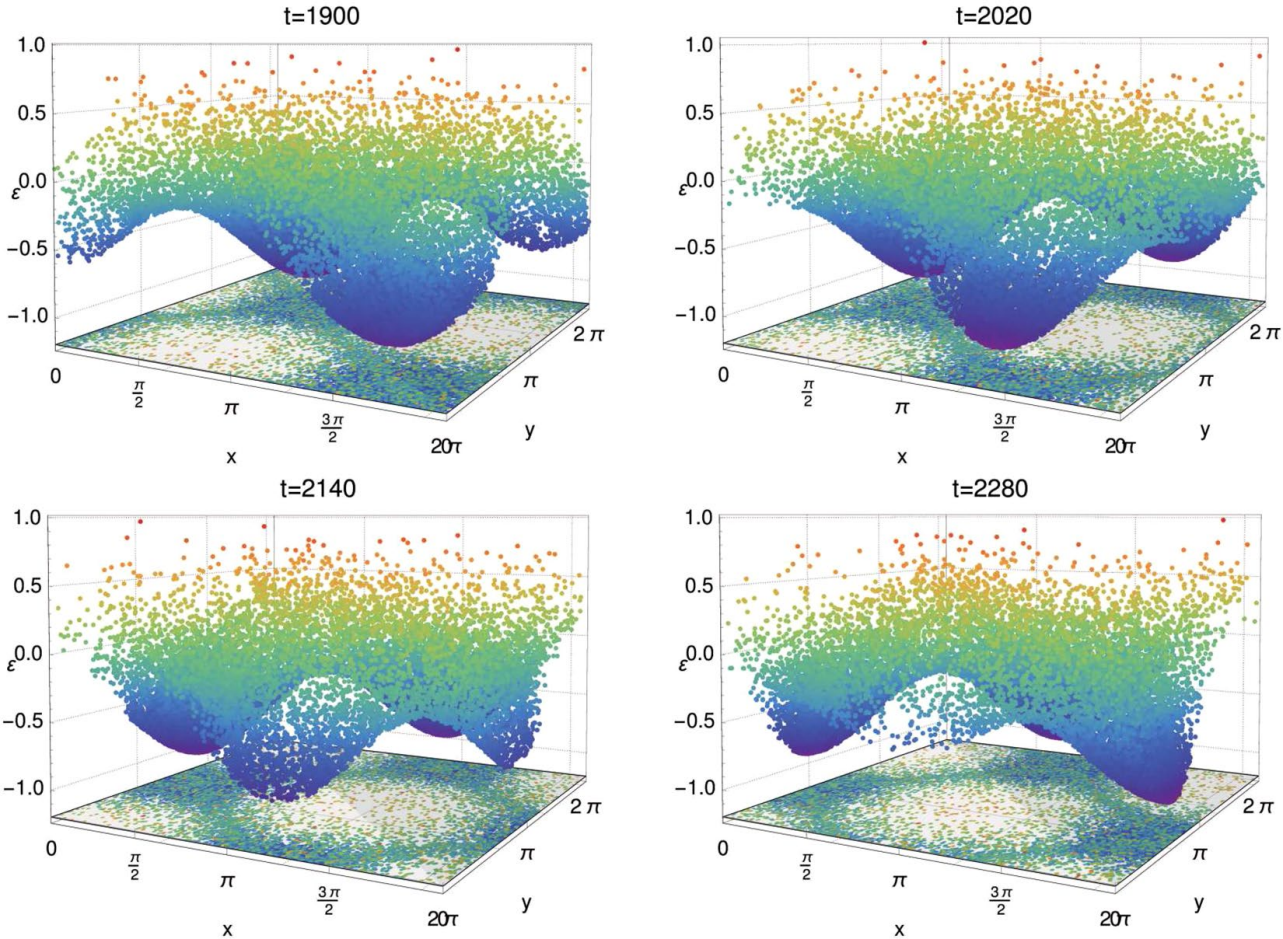
Snapshots taken at different successive times of the positions of the *N* = 25000 particles in the real space during the quasi-stationary state. The *z*-axis represents the particle energy computed by the relation in Eq. (17) and defines the particle color. The low energy particles (LEP) plotted in blue remain trapped in the potential cores, the intermediate energy particles (IEP) may hop from one core to another and the few ‘high’ energy particles (HEP) plotted in yellow and red can move freely throughout the system. The bottom plots are projections on a horizontal plane.

**Figure 3:**
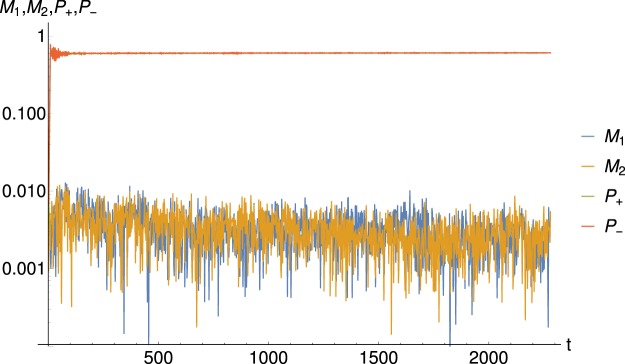
Time behaviour of the mean-fields moduli for the same parameters as in Fig. 2 in linear-log scale. The mean values for mean fields are *N* = 0.0035 and *P*_±_ = 0.6.

Figure 2 shows some results of molecular dynamics symplectic simulations obtained using *N* = 25000 particles and *n* = 1 when the quasi-stationary state (QSS) has been reached. The four panels are snapshots of the real space distributions during the QSS phase. The early time behaviour of the moduli of the mean-fields are represented on Fig. 3. In the QSS regime, the values of the magnetization mean-fields are ${M}_{1}\simeq {M}_{2}\simeq 0.0035$, whereas the polarization mean-fields are ${P}_{+}\simeq {P}_{-}\simeq 0.6$. These values are consistent with the results presented in the Fig. 8 of ref.^18^. The latter shows the linearly unstable and subsequent saturation stages of the time evolution of the moduli of the mean-fields. Because of the symmetry in *x* and *y* in the expression of the Hamiltonian (10) and of the spatial homogeneity, and therefore invariance, in *x* and *y* of the initial conditions, the mean-fields are approximately equal in the *N* ≫ 1 limit, namely (${P}_{+}\approx {P}_{-}={P}_{\pm }$ and ${M}_{1}\approx {M}_{2}=M$). In the three-dimensional plots of Fig. 2, each of the *N* = 25000 particles is represented by a dot as the function of its positions in the 2D [0:2*π*] × [0:2*π*] cell and of its energy, the color of the dot depending on the particle energy. The bottom plots are projections on the horizontal plane that help to visualize the location of the cores, namely the instantaneous location of the crystalline structure. Indeed the cold mixed attractive/repulsive 2*D*-HMF model is a many-body system in which the lowest energy is a state of modulated density: this low-energy fraction of particles defines a crystal in the sense of Landau^28^.

**Figure 4:**
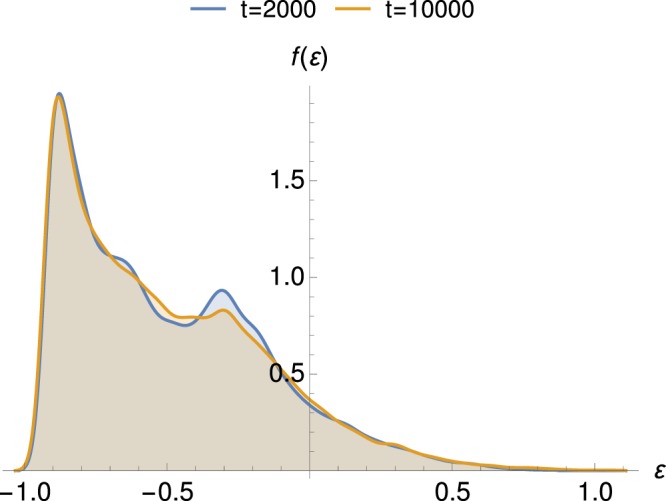
Two histograms representing the energy distribution of particles computed from a symplectic integration with *N* = 2.5 × 10^4^ particles. The energy distributions have been obtained from averaging over the data obtained in 40 equidistant instants in a time range of 80 time units about the times *t* = 2000 and *t* = 10^4^.

Figure 4 shows the energy distribution of particles at two different stages, in the QSS regime and later in the thermalization stage. The energy distributions were obtained from averaging over the data obtained at 40 equidistant instants within a time range of 80 time units about the times *t* = 2000 and *t* = 10^4^.

Considering the particle energy distribution in the QSS regime (about *t* = 2000), it is possible to distinguish three types of particles: i) low energy particles (LEP), appearing in blue on Fig. 2, constituting the majority of the system particles and forming the bulk of the energy distribution function, which stay trapped in the cores of the bicluster structure; ii) a second peak around *ε* = −0.3 reveals a second class of particles constituted by intermediate energy particles (IEP), these particles can hop from one core to another, being still attached to the mean-fields potential, moving coherently in two well defined directions (along the diagonals) and, finally, iii) a few high energy particles (HEP), appearing in red and yellow in the snapshots of Fig. 2, that can move freely in space.

### Potential topology

**Figure 5:**
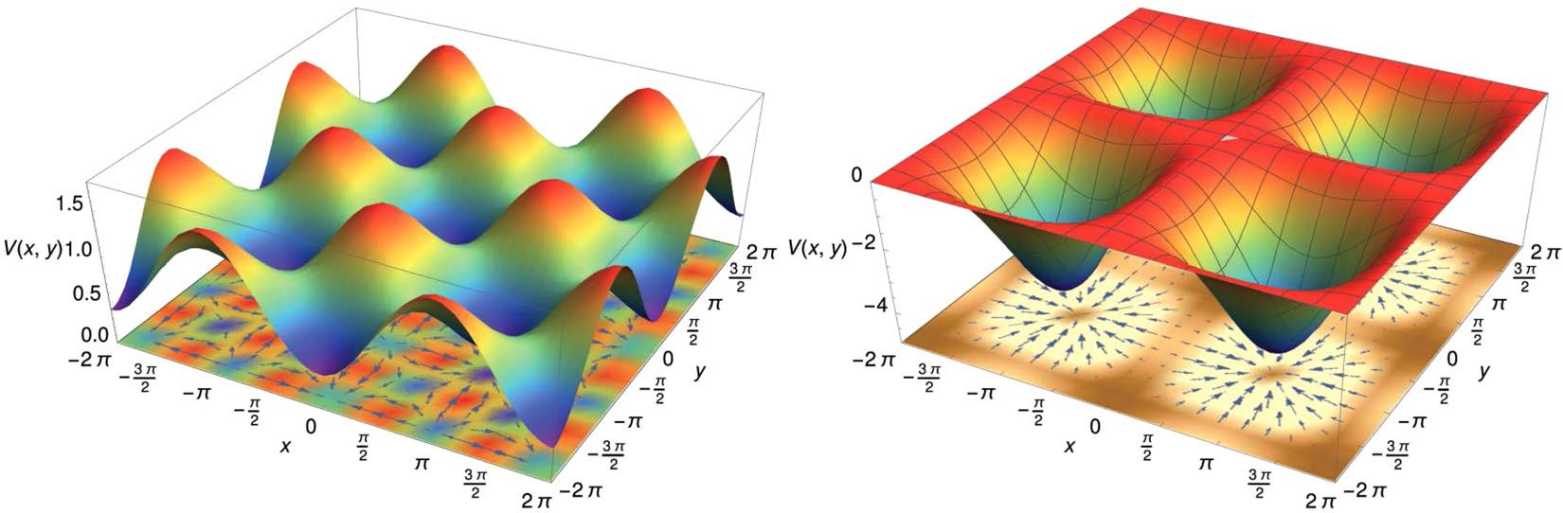
Potential *V*(*x*, *y*) for (left) the mixed attractive/repulsive 2*D*-HMF model, (right) the fully attractive 2*D*-HMF model. The bottom horizontal plots represent the force vector fields $-\,{\boldsymbol{\nabla }}V$.

It is useful to figure out the behaviour of the potential energy associated to the one-particle Hamiltonian (17). This is represented in Fig. 5 in the limit case of the Vlasov limit *N* → *∞* inducing *M*_1_ = *M*_2_ = 0 and for *P*_+_ = *P*_−_ = 0.6. For comparison, the potential associated to the fully attractive 2*D*-HMF model for the same energy density is also represented on the right plot. The bottom plots represent the corresponding force vector fields $-\,{\boldsymbol{\nabla }}V$. The classification of the particles becomes then clear. The particles forming the crystal (LEP) are trapped in the potential wells below the relative extrema of the potential, the free (HEP) particles have their energies larger than that of the absolute extrema of the potential energy and the remaining (IEP) particles are chanelling particles evolving along the diagonals.

### Deviation from thermodynamic equilibrium

Let us now quantify the deviation from thermodynamic equilibrium of the QSS state. A simple way to check whether the system has already reached or is going toward the thermodynamic equilibrium is to compute the reduced kurtosis, *k*, defined as

24 $$k\equiv \frac{\langle {(p-\langle p\rangle )}^{4}\rangle }{{\langle {(p-\langle p\rangle )}^{2}\rangle }^{2}}-3.$$

**Figure 6:**
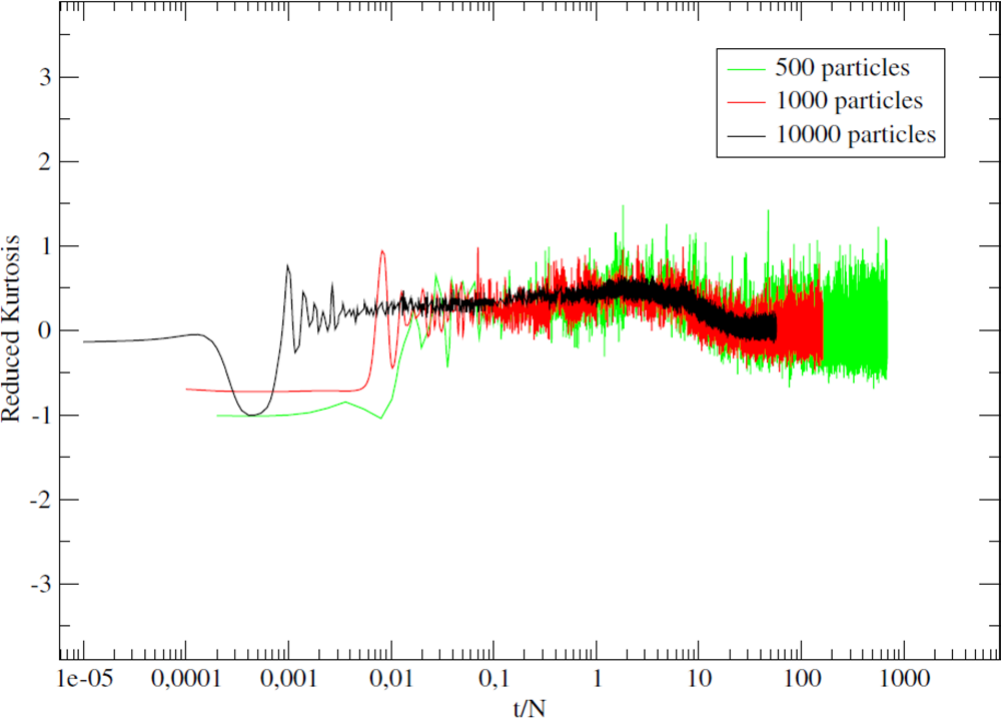
Reduced kurtosis for different system sizes with *N* = 500, 1000 and 10000 particles. The horizontal axis is the time *t* divided by *N* in logarithmic scale. We notice that, as the time increases, *k* approaches and eventually fluctuates about 0. Fluctuations are reduced for larger *N* values. In the *t*/*N* reduced variable, the three curves go to zero simultaneously signaling that the time to reach the thermal equilibrium diverges like *N*.

This is the fourth standardized moment minus 3. For a Gaussian distribution, which characterizes the Maxwell-Boltzmann equilibrium, one has *k* = 0. The graph in Fig. 6 plots the evolution of the reduced kurtosis as a function of the time divided by the number, *N*, of particles. It indicates that the thermalization timescale is proportional to *N*. From this follows, using a proof by contradiction, that the lifetimes of the QSS are, at most, diverging as *N*. Such a scaling would be corroborated by the results of Chavannis^29^.

These are derived from kinetic equations for bidimensional long-range interacting systems and indicate a lifetime for QSSs that scales linearly with *N*. This scaling with *N* is also obtained from a stochastic modeling of the thermalization process involving the disintegration of coherent structures sustaining out-of-equilibrium quasistationary states in the one-dimensional attractive HMF model^6^.

## Combination of Solid and Liquid-Like Features

### Heterogeneous diffusive properties

**Figure 7:**
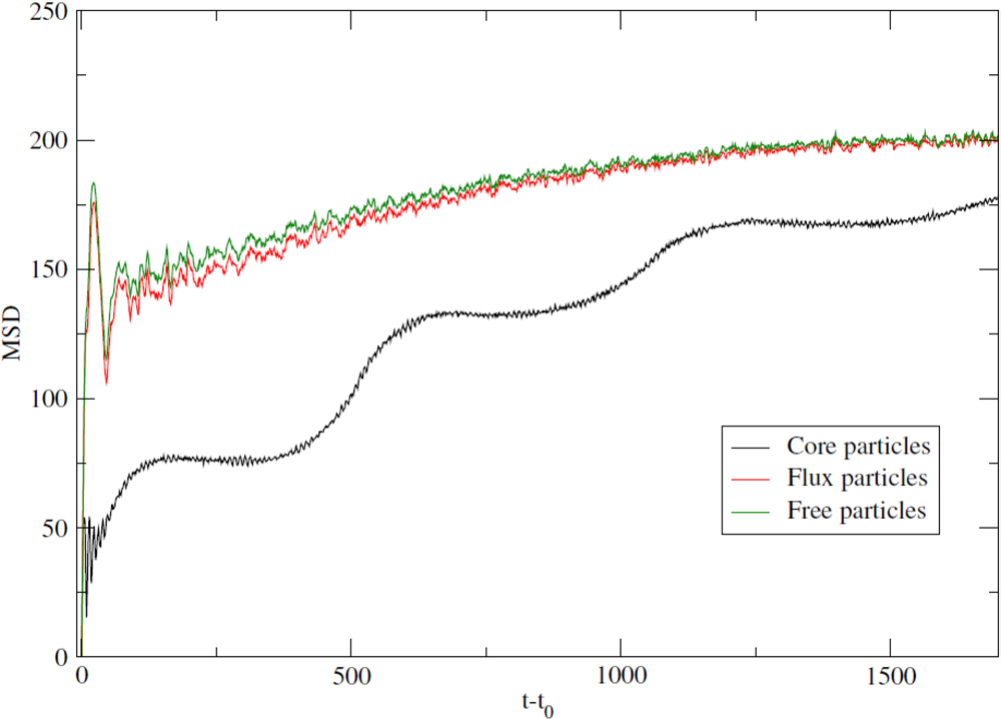
Time evolution of mean square displacement (MSD) in real space for *t*_0_ = 2500, *N* = 10^4^ particles and n = 10. The graph shows the diffusion regime for the three types of particles: in black the LEP, in red the IEP, and in green the HEP. The IEP, which are the particles that form the flux between the cores, diffuse in the same way as the free particles, even if the IEP are trapped to the potential.

The fact that the QSS combines solid and liquid-like features is first evidenced by the heterogeneity of its diffusion properties. Figure 7 presents the computation of the mean square displacement (MSD) for each kind of particles during the QSS regime. The MSD plotted in Fig. 7 is defined as

25 $$\begin{array}{rcl}MSD(t) & = & \frac{1}{N}\sum _{i=1}^{N}\{[{x}_{i}(t)-{x}_{i}({t}_{0}{)]}^{2}+{[{y}_{i}(t)-{y}_{i}({t}_{0})]}^{2}\}\\  & \equiv  & {\langle |{\bf{r}}(t)-{\bf{r}}({t}_{0}{)|}^{2}\rangle }_{N},\end{array}$$

where the brackets ${\langle \cdot \rangle }_{N}$ stands for the average over *N* particles. The results shown were obtained for *t*_0_ = 2500, *n* = 10 and for a total number of 10^4^ particles. The choice for *t*_0_ = 2500 was in order to ensure that the system had already gone into the QSS regime and formed the periodic structure.

The time evolution of the MSD for the core particles shows that they have a vitreous profile, characterized by time intervals with almost no diffusion. This can be interpreted as the Bravais-like lattice having a high viscosity. Meanwhile, the diffusion profile in Fig. 7 and the snapshots of the QSS shown in Fig. 2 show the movement of the cores without changes in the periodic array of the Bravais-like lattice. This global movement of the crystal structure results from the requirement of total momentum conservation so that the heavy cores move slowly in order to counterbalance the rapid movement of the few flux particles.

Conversely, the IEP, which are the particles that support the flux between the cores, and the free particles (HEP) present almost the same profile for diffusion, markedly different from that of the particles (LEP) forming the cores. The diffusion regime for IEP and HEP appears first as diffusive then as sub-diffusive at large time. The latter behaviour is however an artefact due to the fact that the system is confined into a finite square box of sides 2*nπ* by 2*nπ*, in such a way that there exists a maximum value that the mean square displacement can reach. Figure 7 also shows that the flux particles, even trapped to the mean-field potential of the cores, diffuse in a way independent from the vitreous diffusion of the cores. Figure 7 reveals an heterogeneous diffusive behaviour: particles forming the cores of the crystal structure have a glassy behaviour whereas the rest of the particles diffuses normally as in a normal fluid.

**Figure 8:**
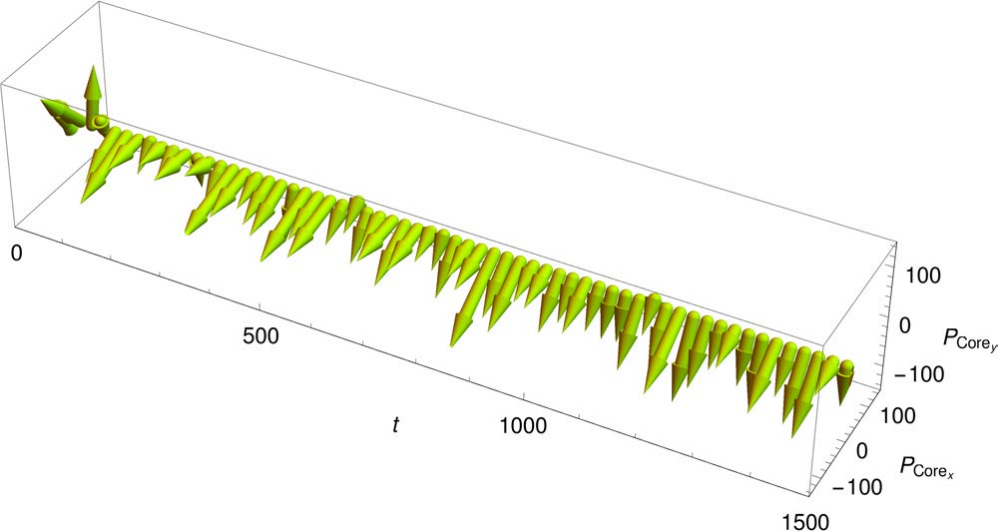
Instantaneous total momentum **P**_Core_ of the particles trapped in the self-consistent potential wells as a function of time for *N* = 25000 particles (same parameters as in Fig. 2). The fraction of trapped particles forming the crystal-like structure remains equal to about 70% during the whole QSS stage for the initial conditions (20) considered in the present study.

Moreover, for the initial conditions under consideration, namely space-homogeneous with vanishing temperature and total momentum, the collective momentum of these diffusive particles flowing through the crystal-like structure is the opposite of the drifting momentum of the clustered particles, namely −**P**_Core_(*t*). The time behaviour of the core total momentum vector has been plotted in Fig. 8. This means that, in the reference frame of the crystal, the particles forming the liquid-like phase have a nonzero mass flow.

## Response to External Perturbations

**Figure 9:**
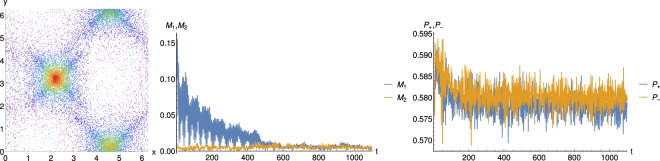
(left) Density plot in the (*x*, *y*) space at the time of the perturbation: the left core is displaced along the *x*-axis by Δ*x* = 0.8; The resulting time behaviours of the mean-fields (center) *M*_1_(*t*), *M*_2_(*t*) and (right) *P*_+_(*t*) and *P*_−_(*t*).

Finally, in order to test the solid character of the QSS, we studied the response of the QSS to external perturbations. A core was displaced at some given time as represented in the left plot of Fig. 9. As visible from the central plot displaying the time behaviors of the magnetization mean-fields, particles from the displaced core snap back into place after a transient oscillatory stage. Indeed, in the case of Fig. 9, all the LEP particles from the left bottom core are displaced at some given time to the right along *x*. This produces an increase of *M*_1_. The core then starts to oscillate along *x*, yet with a decreasing amplitude, about its original position that it recovers at time $t\simeq 500$ which is captured by the damped oscillatory behaviour of *M*_1_. This phenomenology is a solid feature in contrast to a fluid that would be permanently displaced.

## Final Remarks and Paths to Quantum Realization

The results presented here indicate that, in the *N* → ∞ (Vlasov) limit, the mixed attractive/repulsive 2*D*-HMF in the low-temperature regime remains frozen in the QSS having a periodic Bravais-like structure with a flux of particles with non-zero mass flow between the cores. According to previous Monte Carlo results in the microcanonical ensemble^18^, there exists a threshold in the energy density above which the mixed 2*D*-HMF is homogeneous at equilibrium. Moreover former linear theory calculations for the stability of waterbag-like initial conditions^20^ indicate that the homogeneous state will no longer be unstable above some threshold in the initial temperature, forbidding the violent relaxation stage. This leads us to predict the existence of some threshold in the initial temperature above which the QSS reported here does not survive and the system remains quiescent in the homogeneous state.

In the limit of vanishing temperature, that is valid at least in the initial stage of the dynamics considered in this work, the thermal de Broglie wavelength ${\lambda }_{{\rm{th}}}={\mathrm{(2}\pi /(m{k}_{{\rm{B}}}T))}^{\mathrm{1/2}}\hslash$ (with mass *m* = 1 in our model) becomes larger than the interparticle distance and quantum effects are significant. The quantum transposition of this model would therefore be interesting to investigate. In order to prepare for a quantum transposition of the model, mean-field quantum descriptions have been introduced in the Supplementary material. In the case of bosons, a linear theory is presented in the zero temperature limit. This enables to fix the constant parameters in front of the mean-fields in the expression of the potential for which linear instability (violent relaxation) exists in the quantum regime. This secures the existence of QSS, due to the combination of nonlinear effects leading to the saturation of mean-fields and of ergodicity failures for quantum long-range systems^14^. It remains to be studied what is the impact of the nonlinearities entering through quantum effects (in the quantum pressure term) on the nature of the QSS and possibly modify the potential to counteract this effect, if needed. The case of fermions may display the richer phenomenology since, along the attractive direction, one may obtain a transition between a Bose-Einstein condensate with a superfluid phase for strong interaction and a supraconductor state for weak interaction. These last conjectures are left for further studies. Finally, the interaction range involved in the 2*D*-HMF model is infinite. As for the experimental quantum realization, it needs then to involve interactions that have a range longer than that of the dipole interaction having a space dependence as 1/*r*^3^. There is presently a reckless quest in the quantum experimental community to access infinite range interactions. This may be attainable using optical cavities^19^.

Finally, compared with the traditional egg-crate 2D potential represented in the right part of Fig. 5, the potential resulting from the mixed attractive-repulsive interaction, has two barriers in the potential well, one for the confinement of particles forming the crystal-like structure and another one for the IEP fluid-like particles. This offers more freedom to control and access delocalization.

## Electronic supplementary material

Supplementary Information
