# Supplementary material for: Coexisting crystal and liquid-like properties in a 2D long-range self-consistent model
Source: Sci Rep. 2018 Oct 25;8:15800. doi: 10.1038/s41598-018-33889-8 (PMC6202336; doi:10.1038/s41598-018-33889-8)
Supplement: Supplementary file 1 — Supplementary Information [file 41598_2018_33889_MOESM1_ESM.pdf]

Supplementary material for *Coexisting crystal and liquid-like properties in a 2D long-range self-consistent model*

J. M. Maciel[1,2], M. A. Amato [1,3], and M.-C. Firpo[4,*]

[1]Instituto de Física, Universidade de Brasília, CP 04455, 70919-970 - Brasília, Brazil
[2]Campus Paranavaí, Instituto Federal do Paraná, 87703-536 - Paranavaí - PR, Brazil
[3]International Center for Condensed Matter Physics, Universidade de Brasília, CP 04455, 70919-970 - Brasília, Brazil
[4]LPP, CNRS, Ecole Polytechnique, Université Paris-Saclay, PSL Research University, 91128 Palaiseau, France
[*]firpo@lpp.polytechnique.fr

ABSTRACT

The 2D-HMF models involve infinite-range interactions with all-to-all particle couplings. This is a limit case for long-range interactions in which each single particle dynamics is governed by mean-fields, the time evolutions of which depend equally on all particles. In the classical setting, the large-N limit of this model amounts to a Vlasov equation. The objective of this supplementary material is to prepare for a quantum generalization of the 2D-HMF models.

Methods

We shall here introduce some quantum analogues of the $N \to \infty$ description of mean-field models. The Reader is referred to[1] for mathematically rigorous derivations from first principles of such mean-field evolution equations in a general setting. We shall then derive the linear theory of the cold 2D-HMF models in the case where particles are bosons and briefly discuss the case of fermions.

Equations

Let us consider the one-particle Hamiltonian (Eq. 17) and quantize it by the usual rule $\mathbf{p} \mapsto \hbar/i\nabla_{\mathbf{r}}$. If the N particles are undistinguishable quantum particles in the same quantum state, then the system is described by one wave function $\psi(\mathbf{r},t) \in \mathbb{C}$. In the mean-field $N \to \infty$ limit, its evolution is governed by the *mean-field Schrödinger equation*

$$i\hbar\partial_t \psi = -\frac{\hbar^2}{2m}\triangle_{\mathbf{r}}\psi + \left(\int_{\mathbb{R}^2} V\left(\mathbf{r},\mathbf{r}'\right)\rho(\mathbf{r}',t)d\mathbf{r}'\right)\psi, \tag{1}$$

with the density ρ given by

$$\rho(\mathbf{r},t) = |\psi(\mathbf{r},t)|^2 .$$

This model being based on the assumption that all the particles are in the same quantum state, a situation corresponding to Bose-Einstein condensation, is typical for bosons at zero temperature.

In the case of particles having half-integer spin where the wave function is a skew-symmetric function of their positions, that is in the case of fermions, Pauli's exclusion principle applies. Any two fermions cannot be in the same quantum state. In this case, one introduces the density matrix

$$D\left(\mathbf{r},\mathbf{r}',t\right) = \frac{1}{N}\sum_{i=1}^{N}\psi_i(\mathbf{r},t)\,\psi_i^*\left(\mathbf{r}',t\right)$$

that must satisfy the *time-dependent Hartree-Fock equation*[2,3]

$$\begin{aligned} i\hbar\partial_t D\left(\mathbf{r},\mathbf{r}',t\right) &= -\frac{\hbar^2}{2m}\left(\triangle_{\mathbf{r}}-\triangle_{\mathbf{r}'}\right)D\left(\mathbf{r},\mathbf{r}',t\right) \\ &+\int_{\mathbb{R}^2}\left[V\left(\mathbf{r}-\mathbf{r}''\right)-V\left(\mathbf{r}'-\mathbf{r}''\right)\right]D\left(\mathbf{r},\mathbf{r}',t\right)D\left(\mathbf{r}'',\mathbf{r}'',t\right)d\mathbf{r}'' \\ &-\int_{\mathbb{R}^2}\left[V\left(\mathbf{r}-\mathbf{r}''\right)-V\left(\mathbf{r}'-\mathbf{r}''\right)\right]D\left(\mathbf{r},\mathbf{r}'',t\right)D\left(\mathbf{r}'',\mathbf{r}',t\right)d\mathbf{r}''. \end{aligned}$$

We shall now consider the 2D-HMF model at zero temperature in the case where the particles are bosons and form a Bose-Einstein condensate (BEC) described by a wave function $\psi\left(\mathbf{r},t\right)$. In the mean-field $N\to\infty$ limit, its evolution is governed by the mean-field Schrödinger equation (MFSE) (1). The Madelung transformation enables to rewrite the MFSE in the form of fluid equations. Writing

$$\psi\left(\mathbf{r},t\right)=A\left(\mathbf{r},t\right)e^{iS(\mathbf{r},t)/\hbar}$$

with A and S real functions given by

$$A=\sqrt{|\psi|^2}=\sqrt{\rho(\mathbf{r},t)},S=\frac{\hbar}{2i}\ln\left(\frac{\psi}{\psi^*}\right).$$

The velocity field is introduced as

$$\mathbf{v}\left(\mathbf{r},t\right)=\left[\psi^*\left(\mathbf{r},t\right)\nabla\psi\left(\mathbf{r},t\right)-\nabla\psi^*\left(\mathbf{r},t\right)\psi\left(\mathbf{r},t\right)\right]/2mi\rho\left(\mathbf{r},t\right).$$

Injecting this in the MFSE and separating real and imaginary parts yields

$$\frac{\partial\rho}{\partial t}+\nabla\left(\rho\mathbf{v}\right) = 0, \tag{2}$$

$$m\frac{\partial\mathbf{v}}{\partial t}+\nabla\left(V-\frac{\hbar^2}{2m}\frac{\triangle\sqrt{\rho}}{\sqrt{\rho}}+\frac{1}{2}m\mathbf{v}^2\right) = 0. \tag{3}$$

This is the quantum analog of the cold hydrodynamic system of equations introduced in[4]. The irrotational nature of the superfluid flow is inferred from Eq. (3). We shall now derive its linear stability. Considering the following expression of the potential (up to constant terms) as

$$V(x,y,t)=-c_1M_1\cos(x-\psi_1)-c_2M_2\cos(y-\psi_2)-\frac{d}{2}[P_+\cos(x+y-\psi_+)+P_-\cos(x-y-\psi_-)],$$

and proceeding as in[4], yields basically the same eigenfrequencies as in the classical case with quantum additional terms, meaning that classical results are recovered by canceling $\hbar$ terms. Indeed, as for the $m=\pm1,l=\pm1$ modes, we have

$$\omega^2=-\frac{1}{2}(d-2\hbar^2), \tag{4}$$

for the $m=\pm1,l=0$ modes

$$\omega^2=-\frac{1}{2}(c_1-\frac{\hbar^2}{2}), \tag{5}$$

and, for the $m=0,l=\pm1$ modes

$$\omega^2=-\frac{1}{2}(c_2-\frac{\hbar^2}{2}). \tag{6}$$

As for the other modes, their eigenfrequencies are zero in the classical frame. In the present quantum version with cold bosons, quantum effects introduce waves with eigenfrequencies

$$\omega_{m,l}=(m^2+l^2)\hbar/2. \tag{7}$$

The above results indicate that quantum effects act to stabilize the spatially homogeneous initial state and give the conditions on the choice of the parameters c_1, c_2 and d ensuring that the violent relaxation process observed in the classical mixed attractive-repulsive case still exists in the quantum frame.

We shall eventually introduce the *mean-field Gross-Pitaevskii equation* (MFGPE)[5]

$$i\hbar\partial_t\psi = -\frac{\hbar^2}{2m}\triangle_{\mathbf{r}}\psi + \left(\int_{\mathbb{R}^2} V\left(\mathbf{r},\mathbf{r}'\right)\rho(\mathbf{r}',t)d\mathbf{r}' + \mathscr{V}(\rho)\right)\psi, \tag{8}$$

with $\mathscr{V}(\rho)$ some potential depending on the density. Using the Madelung transformation for the wave function, the MFGPE is equivalent to the system of hydrodynamic equations

$$\frac{\partial\rho}{\partial t} + \nabla(\rho\mathbf{v}) = 0, \tag{9}$$

$$m\frac{\partial\mathbf{v}}{\partial t} + \nabla\left(V + \mathscr{V} - \frac{\hbar^2}{2m}\frac{\triangle\sqrt{\rho}}{\sqrt{\rho}} + \frac{1}{2}m\mathbf{v}^2\right) = 0. \tag{10}$$

These equations have the structure of the dynamic equations of superfluids at zero temperature. In the case of a Bose Einstein condensate, the additional potential $\mathscr{V}(\rho)$ contains the information on short-range interaction (binary collisions) that was absent from the MFSE. In the case of fermions having the same probability distribution, forming a mixture of N pure states, this may model the Fermi pressure, that exists even at zero temperature. This follows from the Pauli exclusion principle forbidding any two fermions with the same spin to occupy the same quantum state within a quantum system simultaneously. This Fermi pressure[6] opposes any attempt of compression of the fermionic gas.

References

1. Golse, F. The Mean-Field Limit for the Dynamics of Large Particle Systems, Journées équations aux dérivées partielles, Forges-les-Eaux (2003) (and refs. therein).

2. Bardos, C., Golse, F., Gottlieb, A. & Mauser, N. Mean field dynamics of fermions and the time-dependent Hartree-Fock equation *J. Math. Pures Appl.* (9), **82** (2003), pp. 665-683

3. Benedikter, N., Porta, M. & Schlein, B. Mean-Field Evolution of Fermionic Systems *Communications in Mathematical Physics* **331**:3, 1087-1131 (2014).

4. Maciel, J. M., Firpo, M.-C. & Amato, M. A. Some statistical equilibrium mechanics and stability properties of a class of two-dimensional Hamiltonian mean-field models *Physica A* **424**, 34-43 (2015).

5. Dalfovo, F., Giorgini, S., Pitaevskii, L. P. & Stringari, S. Theory of Bose-Einstein condensation in trapped gases *Rev. Mod. Phys.* **71**, 463 (1999).

6. Chevy, F. & Salomon, C. Les gaz de fermions ultra-froids *Images de la physique* (CNRS), p. 90-97 (2005).
